# IMPLICATION OF SLEEPING DURATION ON THE OVERALL HEALTH STATE OF FAMILY CAREGIVERS

**DOI:** 10.1093/geroni/igac059.2356

**Published:** 2022-12-20

**Authors:** Babatope Ogunjesa, Andiara Schwingel, Tosun Ozgur, Alice Oloo

**Affiliations:** University of Illinois--Urbana-Champaign, Champaign, Illinois, United States; University of Illinois, Urbana Champaign, Urbana Champaign, Illinois, United States; Near East University, Nicosia, Nicosia, Cyprus; University of Illinois, Urbana Champaign, Urbana Champaign, Illinois, United States

## Abstract

Family caregiving is integral to long-term care for older adults. The National Alliance for Caregiving and AARP Public Policy Institute in 2020 estimated there are 53 million Americans providing care for loved ones. Caregiving intensity often impacts the health and well-being of caregivers, including influencing their sleep duration. The Centers for Disease Control and Prevention (CDC) recommended an average of 7 or more sleeping hours for adults. Unfortunately, most caregivers' average hours of sleep per night do not meet this recommendation. Using secondary data from the Long-Term Caregiving study conducted by the AP-NORC Center for Public Affairs Research, the present study establishes the effect of sleep duration on the overall health of family caregivers while adjusting for socio-demographic factors. A binary logistic analysis was conducted to infer the effects of sleep duration, age, gender education, race, employment status, and household income relative to reporting high or low overall health wellbeing. The binary logistic model was statistically significant, χ2(16) = 77.5, p < .0001, Nagelkerke R2=0.111. Family caregivers with a sleeping duration of 7 hours or more were twice as likely to report a high overall health status than those who slept less than 7 hours (OR=2.08, 95%CI [1.52 – 2.84]). Increasing household income and being employed were positively associated with high overall health status. We do not find a statistically significant effect of gender, education level, age group, and race on overall health status. We recommend that policies that support family caregivers at a micro and macro level be implemented.

